# Diagnostic and Management Challenges of Caesarean Scar Ectopic Pregnancy in a Lower Middle Income Country

**DOI:** 10.1155/2019/4257696

**Published:** 2019-11-11

**Authors:** Kwaku Asah-Opoku, Nana Essuman Oduro, Alim Swarray-Deen, Kareem Mumuni, Isaac Osei Koranteng, Rita Catherine Senker, Marcus Rijken, Kobinah Nkyekyer

**Affiliations:** ^1^Department of Obstetrics and Gynecology, University of Ghana School of Medicine and Dentistry (UGSMD), Korle-Bu, Accra, Ghana; ^2^Korle-Bu Teaching Hospital, Department of Obstetrics and Gynaecology, Korle-Bu, Accra, Ghana; ^3^Department of Obstetrics and Gynaecology, University Medical Centre Utrecht, Utrecht University, Utrecht, Netherlands; ^4^Julius Global Health, Julius Centre for Health Sciences and Primary Care, University Medical Centre Utrecht, Utrecht University, Utrecht, Netherlands

## Abstract

Caesarean scar ectopic pregnancy is defined as the implantation of the blastocyst in a previous Caesarean scar. It is a rare type of ectopic pregnancy. The incidence is however rising due to the increasing rates of Caesarean sections as well as in-vitro fertilization embryo-transfer. It can be diagnosed early by ultrasound. This remains a challenge in lower middle income countries where the availability of high resolution ultrasound and the skill for such sonography may be lacking. Misdiagnosis or a delay in diagnosis often leads to poor treatment outcomes. We present a case of a gravida 3 para 2 + 0 who had laparotomy for a caesarean scar pregnancy and highlight the challenges associated with diagnosis and management of this rare ectopic pregnancy in a lower middle income country.

## 1. Introduction

Caesarean scar ectopic pregnancy or simply Caesarean scar pregnancy (CSP) refers to a gestational sac that is implanted wholly or partially within a previous Caesarean section (CS) scar. It was first reported in 1978 [1, 2]. Incidence rates of 1/1800–1/2500 pregnancies have been documented [3, 4]. Its incidence is increasing due to increasing rates of CS and in-vitro-fertilization embryo- transfer [5, 6]. Various mechanisms have been proposed as pathophysiology of the CSP but the main mechanism is the invasion of a microscopic tract within the CS scar by the blastocyst as it implants [7]. Fibrosis and poor wound healing are responsible for the formation of the defect in the wound [3]. High income countries have easy access to high resolution ultrasound and magnetic resonance imaging compared to lower middle income countries(LMICs) enabling prompt diagnosis and treatment of CSP in these high income countries.

## 2. Case Presentation

**Figure 1:**
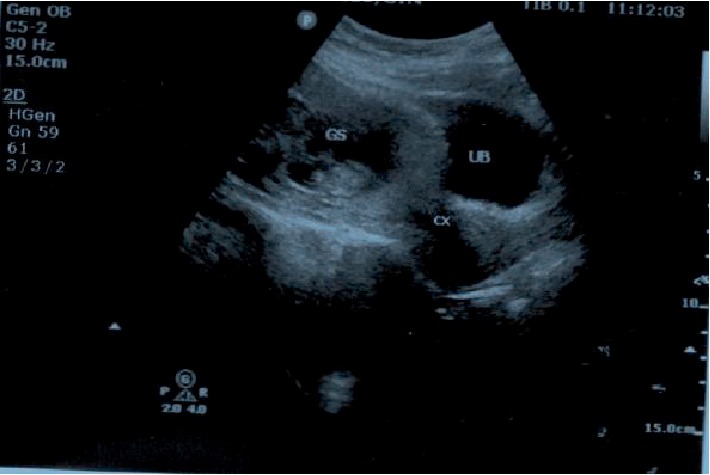
An empty endocervical canal and a gestational sac in the lower uterine segment with a ballooned out lower uterine segment, GS = Gestational sac, Cx = Cervix, UB = Urinary bladder.

**Figure 2:**
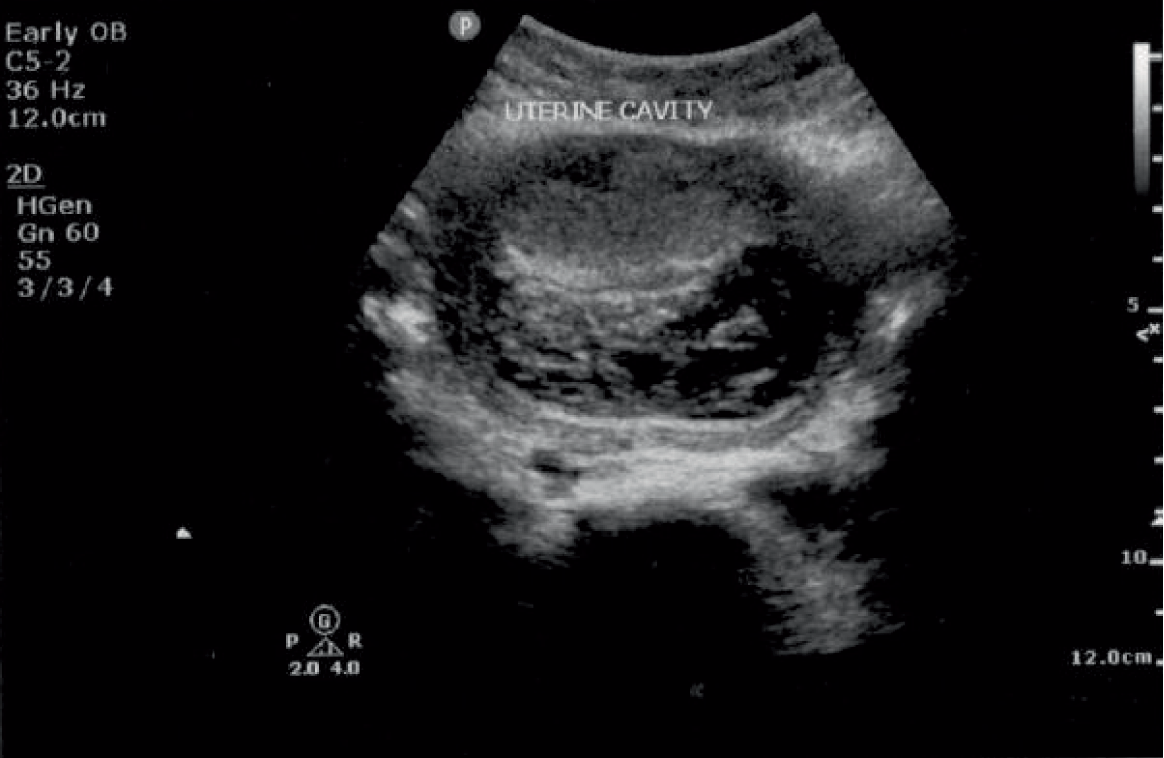
Uterine cavity with some clots.

**Figure 3:**
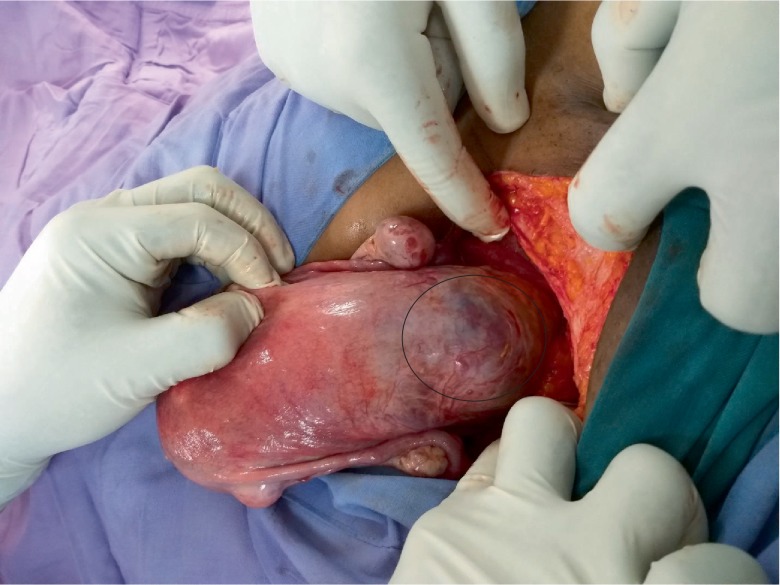
Thinned and ballooned out lower uterine segment.

**Figure 4:**
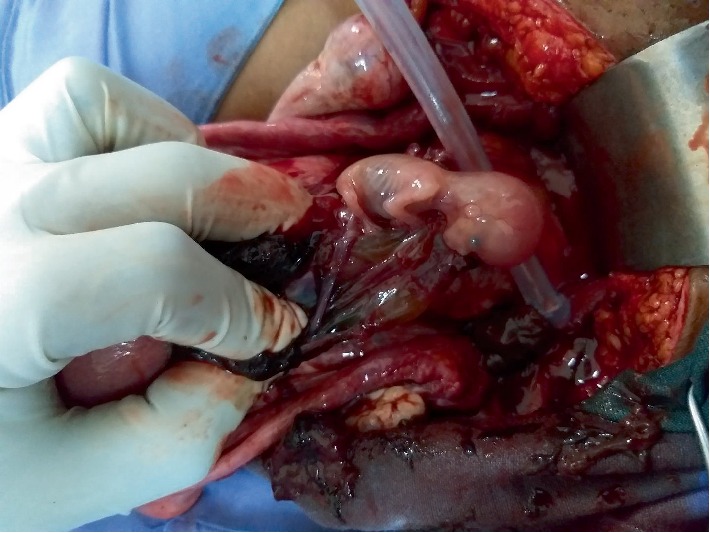
Conceptus and trophoblastic tissue after incision was made through the lower uterine segment.

**Figure 5:**
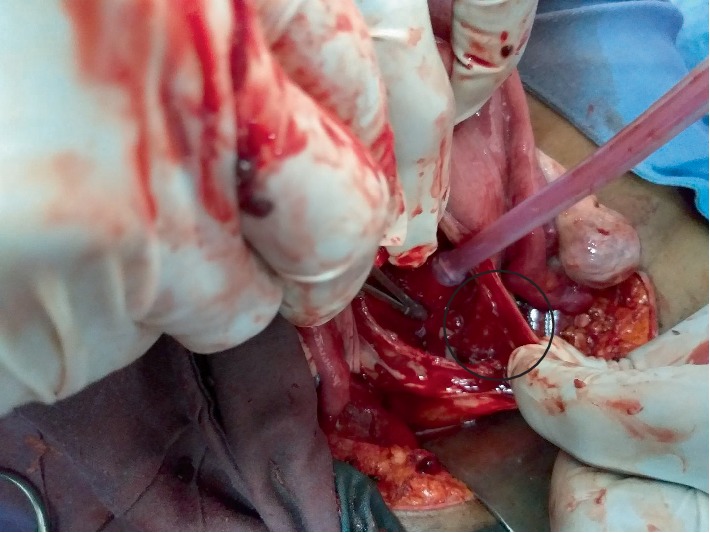
The thinned out Caesarean section scar and bleeding from the base of the trophoblastic tissue attachment to the previous Caesarean section scar.

**Figure 6:**
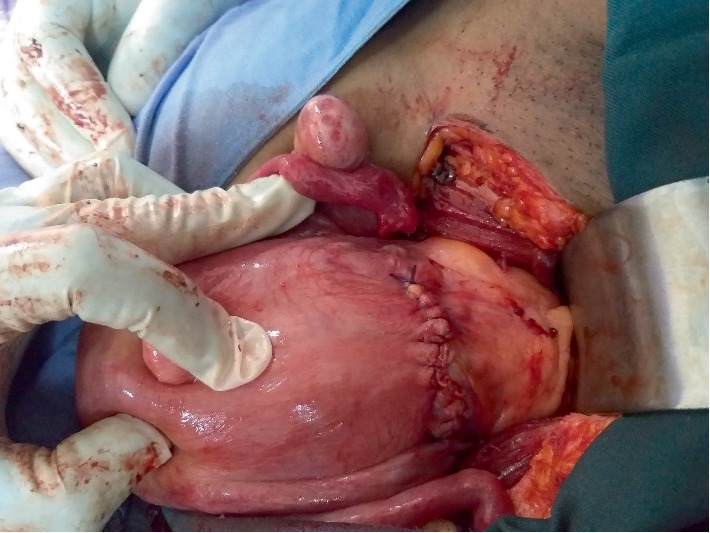
The hysterotomy wound repaired in two layers.

A 38-year-old gravida 3 para 2 presented with scanty vaginal bleeding and mild lower abdominal pain in early pregnancy. Her last menstrual period was two months prior to presentation. She conceived naturally and the pregnancy had been uneventful until her presentation. She had had two previous CS: the first was planned for primigravida breech presentation and the second was performed for fetal distress in a woman with previous CS. She had no significant medical history. On clinical examination, she looked generally well with normal general, cardiovascular, respiratory and abdominal findings. On vaginal examination, there was some scanty blood at vaginal introitus. A sterile speculum exams showed a normal looking cervix with scanty blood at the external cervical os. A bimanual examination showed an anteverted uterus which was bulky. The external cervical os was closed with no adnexal tenderness or cervical excitation tenderness. A urine pregnancy test was positive and therefore this patient was managed as a case of threatened abortion. An appointment for clinical review and pelvic ultrasound was scheduled at the outpatient department (OPD) the next day. The scan showed a singleton intrauterine pregnancy with a gestational age of 7 weeks 4 days. The bleeding per vaginam had subsided and therefore she was scheduled for review in a week. However, five days after this OPD visit, she presented to the gynaecology emergency room with repeat minimal bleeding per vaginam and mild lower abdominal pain. Her cervical os remained closed and a repeat pelvic ultrasound scan with an abdominal probe showed a bulky uterus with a gestational sac that appeared to be within the endocervical canal. A sonographic impression of cervical ectopic pregnancy was made. She was admitted and the laboratory investigations showed haemoglobin level of 12.6 g/dl, her WBC = 7.19 × 10^9^/L and platelet level 288 × 10^9^/L. Her serum beta-HCG level was 105,000 mIU/ml. Her liver function tests and blood urea electrolytes and creatinine were normal. She had a repeat abdominal ultrasound scan 2 days into admission. This scan was done in combination with a transvaginal ultrasound. The findings were a bulky uterus with a gestational sac that appeared to be within the endocervical canal but on transvaginal interrogation, the gestational sac appeared to be at the upper part of the endocervix above the isthmus and in close proximity to the endometrial cavity. The chorionic tissue appeared to cover the internal os. No adnexal mass or pouch of Douglas mass was seen. The impression was a low implantation pregnancy likely to be a developing placenta praevia or a cervical pregnancy at 9 weeks. She was managed as a case of cervical ectopic pregnancy at this point and she received 50 mg of intramuscular methotrexate and had a repeat serum beta HCG done 3 days after, which showed a level of 106,730 mIU/ml. A repeat pelvic ultrasound showed a gestational sac containing single viable fetal pole in the lower segment of the uterus bulging into the bladder (Figure 1). The myometrial wall thickness between the gestational sac and the bladder was 2 mm. There was accumulation of hypoechoeic fluid in a lattice appearance suggestive of clots behind the gestational sac (Figure 2). The endocervical canal was empty (Figure 1). A sonographic diagnosis of unruptured Caesarean section scar ectopic was made. She was counselled for exploratory laparotomy same day and had dissection into the thinned out uterine wall through the Caesarean section scar, removal of the gestational sac and use of figure of eight stitches to secure haemostasis at the site of attachment of the gestational sac. The dissection invariably ended up as a hysterotomy which was repaired after evacuation of the conceptus (Figure 3). The findings were a fourteen week size uterus with a bulge in the lower portion (Figure 4). There was a gestational sac approximately one centimeter from the internal cervical os of the uterus embedded in the myometrial wall with retroplacental clots of approximately 400 ml (Figures 5 and 6).There was thinning out of the wall of the lower uterine segment (Figure 6). She had normal fallopian tubes and ovaries and the total blood loss was approximately 800 ml. Her recovery was uneventful and she was discharged home on the third postoperative day.

## 3. Discussion

CSP is one of the rarest types of ectopic pregnancy. It occurs when the blastocyst implants on the Caesarean scar [1].

Various risk factors have been identified as increasing the chances of a woman developing a CSP. The number of previous CS does not correlate with the risk for a CSP [8]. However, women who have had an elective CS for breech presentation in a previous pregnancy are the ones mostly at risk due to poor formation of the lower uterine segment [8]. This patient's first CS was on account of a breech presentation at term and put her at a higher risk. This history should raise a higher suspicion of a CSP as a cervical pregnancy has been found to be very uncommon in women with a previous CS [9].

Women with CSP often present with slight vaginal bleeding with mild abdominal discomfort [10] as was the case in this presentation. The diagnosis is usually made by a high resolution pelvic ultrasound, a transvaginal ultrasound or a magnetic resonance imaging technique. A combination of the transabdominal and transvaginal ultrasound scanning procedures have been shown to have a higher accuracy than the transabdominal or transvaginal ultrasound used alone [11]. CSP diagnosis with an ultrasound may pose some diagnostic challenges and a high index of suspicion is required to make an accurate diagnosis of CSP. It is not surprising therefore, that three different transabdominal ultrasound scans and a transvaginal ultrasound were done in this patient before the diagnosis of CSP was finally made. The sonographic criteria for CSP include an empty uterine cavity and closed and empty cervical canal, a gestational sac that is implanted in the previous CS scar, a gestational sac that fills the niche of the scar, a thin or absent myometrial layer between the gestational sac and the bladder, yolk sac, embryo and cardiac activity may or may not be present, evidence of functional trophoblastic circulation on colour flow Doppler and the negative ‘sliding organs' sign [9]. The absence of myometrium between the gestational sac and the bladder and an empty uterine cavity as well as an empty cervical canal helps to distinguish cervical ectopic pregnancy from a CSP [12, 13]. There were challenges with accurate ultrasound diagnosis of the CSP in the earlier three ultrasound scans. CSP was not diagnosed in this patient with the earlier ultrasound scans mainly because of the low index of suspicion in the patient. The challenge with diagnosis of CSP could also be because of the fact that because it is rare, most of the Obstetricians and Gynaecologists and sonographers may not have encountered it previously in their ultrasound experience. Indeed the challenges of diagnosing CSP and misdiagnosis of CSP have been highlighted in previous publications [14, 15]. The final ultrasound however that was done in this patient showed the uterine cavity with some clots of blood (Figure 2) and an empty cervical canal (Figure 1), a gestational sac implanted in the previous CS scar with the gestational sac containing a single viable fetal pole in the lower segment bulging into the bladder(Figure 1). There was a thin myometrial wall thickness of 2 mm between the gestational sac and the bladder (Figure 1). Reduction in CS rates by reducing unnecessary CS will help to reduce the rates of CSP. In an era of increasing CS, emphasis should be made on the diagnosis of CSP in women with a history of CS during training and refresher courses in ultrasonography for Obstetrician Gynaecologists and sonographers.

There are various modalities for the management of CSP. These include expectant management, intramuscular or intra-lesional injection of methotrexate and surgical treatment. Surgical treatment is associated with reduced risk of uterine rupture and torrential bleeding from the CSP [16]. That was why it was employed for management of this patient. Expectant management of CSP has a high risk of uterine rupture and maternal death. With the thinned out myometrial tissue at the site of the previous CS scar, an attempt at dilatation and curettage or manual vacuum aspiration could have resulted in incomplete evacuation, bladder injury or uterine rupture along the previous CS scar and increased the morbidity and mortality associated with the management of this case. Laparoscopic and endoscopic management of CSP have been described [17] but the skill and equipment needed for it in our center were not available.

In future pregnancies, the risk of recurrence of CSP is higher [9]. There is also an increased risk of morbidly adherent placenta. There is the need to educate women at risk about the need to report early when pregnant. There is therefore the need for early ultrasound scan in the subsequent pregnancies to rule out recurrence [9]. This was communicated to the patient before she was discharged. Most reports have advocated a CS in the next pregnancy to reduce the risk of uterine rupture and to also enhance adequate closure of the incision in the lower uterine segment [9].

## 4. Conclusion

The diagnosis and management of CSP can be challenging in LMICs and a high index of suspicion is required to allow for early diagnosis and management to reduce the morbidities and mortalities associated with it. Transvaginal ultrasound scan is required in early pregnancy for women with a previous CS scar where the suspicion of CSP is high. Provision of high resolution ultrasound scans and training and refresher courses that highlight the diagnosis of CSP in a world with increasing rates of Caesarean sections will help in early diagnosis of CSP.
